# Sequence analysis of dolphin ferritin H and L subunits and possible iron-dependent translational control of dolphin ferritin gene

**DOI:** 10.1186/1751-0147-50-42

**Published:** 2008-10-27

**Authors:** Azusa Takaesu, Kiyotaka Watanabe, Shinji Takai, Yukako Sasaki, Koichi Orino

**Affiliations:** 1 Laboratories of Veterinary Biochemistry, School of Veterinary Medicine, Kitasato University, Aomori 034-8628, Japan; 2 Animal Hygiene, School of Veterinary Medicine, Kitasato University, Aomori 034-8628, Japan

## Abstract

**Background:**

Iron-storage protein, ferritin plays a central role in iron metabolism. Ferritin has dual function to store iron and segregate iron for protection of iron-catalyzed reactive oxygen species. Tissue ferritin is composed of two kinds of subunits (H: heavy chain or heart-type subunit; L: light chain or liver-type subunit). Ferritin gene expression is controlled at translational level in iron-dependent manner or at transcriptional level in iron-independent manner. However, sequencing analysis of marine mammalian ferritin subunits has not yet been performed fully. The purpose of this study is to reveal cDNA-derived amino acid sequences of cetacean ferritin H and L subunits, and demonstrate the possibility of expression of these subunits, especially H subunit, by iron.

**Methods:**

Sequence analyses of cetacean ferritin H and L subunits were performed by direct sequencing of polymerase chain reaction (PCR) fragments from cDNAs generated via reverse transcription-PCR of leukocyte total RNA prepared from blood samples of six different dolphin species (*Pseudorca crassidens*, *Lagenorhynchus obliquidens*, *Grampus griseus*, *Globicephala macrorhynchus*, *Tursiops truncatus*, and *Delphinapterus leucas*). The putative iron-responsive element sequence in the 5'-untranslated region of the six different dolphin species was revealed by direct sequencing of PCR fragments obtained using leukocyte genomic DNA.

**Results:**

Dolphin H and L subunits consist of 182 and 174 amino acids, respectively, and amino acid sequence identities of ferritin subunits among these dolphins are highly conserved (H: 99–100%, (99→98) ; L: 98–100%). The conserved 28 bp IRE sequence was located -144 bp upstream from the initiation codon in the six different dolphin species.

**Conclusion:**

These results indicate that six different dolphin species have conserved ferritin sequences, and suggest that these genes are iron-dependently expressed.

## Background

Ferritin is a ubiquitous iron storage protein found in all living organisms [1-3]. It also functions to segregate iron in a non-toxic form to prevent iron-catalyzed reactive oxygen species production [3-7]. Mammalian tissue ferritins consist of two functionally different subunits, termed as H (heavy chain, heart-type) and L (light chain, liver-type), and a central cavity accommodating up to 4,500 iron atoms [1,8]. A novel mitochondrial ferritin has also found in humans and rodents, and their ferritins only consist of a novel H-type subunit with 80% identity to their H subunits [9]. The H subunit plays a crucial role in incorporating iron through its ferroxidase activity [10,11]. The L subunit lacks ferroxidase activity, but is involved in iron nucleation to allow more iron to be sequestered [8,11-13].

The H and L subunits of many mammalian ferritins have been sequenced; each subunit shows high amino acid sequence identity with the corresponding subunit of other species despite there being low identity (50–56%) between the H and L subunits within species [2,3,7,14]. Amino acids associated with the ferroxidase center are perfectly conserved in all known mammalian H subunits, and the residues involved in iron nucleation are highly conserved in all known mammalian L subunits [2,3,7,8,14].

Translation of both H and L subunits of mammalian ferritins is iron-dependently regulated through the interaction of the iron-responsive element-binding protein (IRE-BP) with the IRE found in the 5'-untranslated region of ferritin mRNAs [3,15]. Ferritin H and L subunits are also transcriptionally activated by oxidative stress through the antioxidant-reactive element (ARE), a region far upstream of the 5'-untranslated region of H and L subunit genes [16,17].

Although amino acid sequences of mammalian ferritin H and L subunits were high conserved over species, full sequence analysis of marine mammal ferritin has not yet been performed. This study reveals ferritin coding sequences from six different dolphin species (*Pseudorca crassidens*, *Lagenorhynchus obliquidens*, *Grampus griseus*, *Globicephala macrorhynchus*, *Tursiops truncatus*, and *Delphinapterus leucas*). Sequence analysis of the 5'-untranslated region of the H subunit gene also provides strong evidence of iron-dependent ferritin expression through an IRE.

## Materials and methods

### Preparation of total RNA and genomic DNA from blood samples

Blood samples were obtained from the tail veins of apparently healthy individuals of six different species of dolphin (*P. crassidens*, *L. obliquidens*, *G. griseus*, *G. macrorhynchus*, *T. truncatus*, and *D. leucas*) kept at Yokohama Hakkeijima Sea Paradise (Yokohama, Japan). Leukocytes were prepared from heparinized blood samples by centrifuging at 1,700 × g for 20 min. Preparation of total RNA and genomic DNA from leukocytes was carried out using the RNeasy midi kit (Qiagen Inc., CA, USA) and QIAamp DNA Blood midi kit (Qiagen Inc.), respectively.

### Reverse Transcription-polymerase chain reaction (RT-PCR) and cloning

First-strand cDNA was synthesized using total RNA (500–1,000 ng) in 20 μl of reaction buffer containing 10 units of transcriptor reverse transcriptase (Roche Diagnostics GmbH, Mannheim, Germany), dNTPs (20 nmol each), and oligo(dT) primer (20 pmol). PCR was performed in reaction buffer (25 μl) containing 1 μl of the above first-strand cDNA, PrimeSTAR HS (Premix, 1×) (TaKaRa Bio Inc., Shiga, Japan), and 25 or 2.5 pmol each of sense and antisense primers. The sense and antisense primers were as follows: H sense: 5'-CCCGCCGCCGCTCCAGCGCCGCGCAGCC-3'; H antisense: 5'-GGTAAAGGAAACCCCAACATGCATGCA-3'; L sense: 5'-ATGAGCTCCCAGATTCGTCAGAATTATT-3'; L antisense: 5'-CTAGTCGTGCTTGAGGGTAAGCCTTTCG-3'. In addition, for direct sequencing of PCR fragments, sense and antisense primers were designed to contain the coding sequence, including the 5'- and 3'-untranslated regions of the L subunit, from nucleotide sequences of known mammalian L subunits [14,18,19]. Sense and antisense primers were 5'-CGGGACCAGCCACCATTTTTTAACTCCT-3' and 5'-TTTCCATATGGTCCAAGGCTT-3', respectively. Reaction conditions were 2 min at 98°C followed by 35 cycles of 10 sec at 98°C, 30 sec at 65°C, and 60 sec at 72°C. The PCR fragment (approximately 600 bp) was excised and purified from a 3% agarose gel using the MinElute Gel Extraction Kit (Qiagen) and cloned into Zero-blunt TOPO plasmid (Invitrogen Life Technologies, Tokyo, Japan) for transformation of *E. coli *cells. The cloned fragment was sequenced in both directions using appropriate primers by an automated ABI PRISM 310 Genetic Analyzer (Applied Biosystems, Tokyo, Japan).

### Sequencing of IRE of H subunit gene

Sequence analysis of the IRE and its flanking region was performed using dolphin leukocyte genomic DNA. Sense and antisense primers were designed based on the human H subunit gene (AF139813): sense primer: 5'-CGGGGCGGGCGGCGCTGATTGGCCGGGG-3'; antisense: 5'-CATGGACAGGTAGACATAGGAGGCGTAG-3'. PCR conditions were the same as described above except using genomic DNA (100–200 ng). After electrophoresis, PCR fragments were purified from the gel and used for direct sequencing as described above.

### Sequence analysis

Amino acid sequences were deduced from nucleotide sequences determined by cDNA sequencing and direct sequencing of PCR fragments. Sequence analyses were performed using the computer software GENETYX-MAC (Soft Development Co. Ltd., Tokyo, Japan). Secondary structural prediction of the IRE and its flanking sequences was also performed using GENETYX-MAC.

## Results

### Sequence analysis of dolphin ferritin H and L subunits

**Figure 1:**
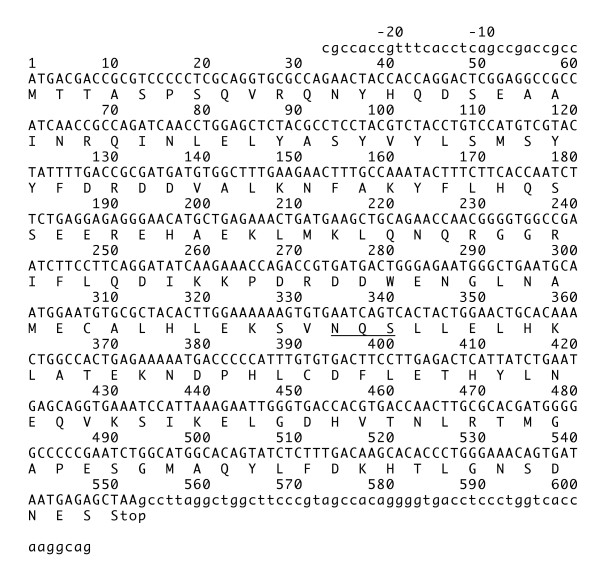
**Nucleotide and deduced amino acid sequences of ferritin H subunit cDNAs from dolphin (*P. crrassidens*) leukocytes.** The sequence contains the coding (capital letters) and noncoding (lower-case letters) regions. Amino acids underlined represent the putative glycosylation site. This sequence data has been submitted to the DDBJ/GenBank/EMBL nucleotide sequence database under accession number AB371409.

**Figure 2:**
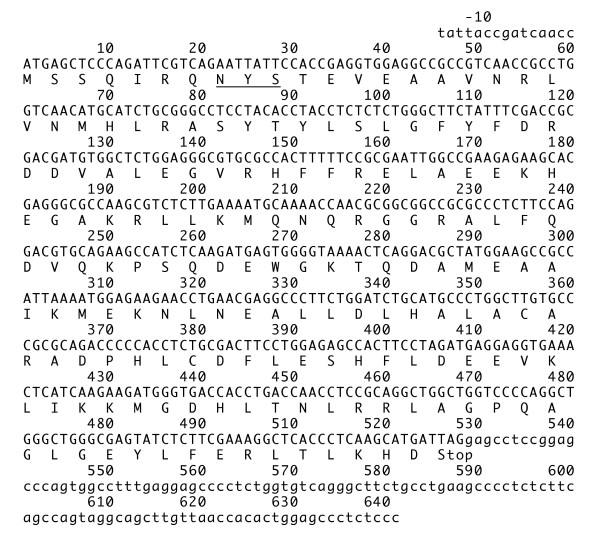
**Nucleotide and deduced amino acid sequences of the ferritin L subunit of dolphin (*P. crrassidens*) leukocytes. **The sequence contains the coding (capital letters) and noncoding (lower-case letters) regions. Amino acids underlined represent the putative glycosylation site. This sequence data has been submitted to the DDBJ/GenBank/EMBL nucleotide sequence database under accession number AB371411.

**Figure 3:**
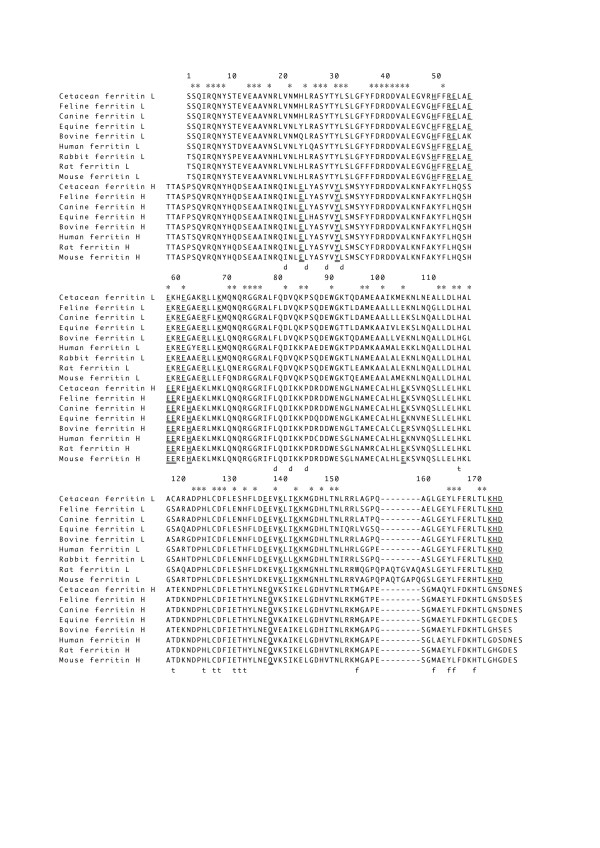
**Alignment of mammalian ferritin H and L subunits.** The amino acid numbering is derived from mammalian L subunit sequences, but does not include the additional octapeptide. Both H and L subunits contain residues associated with dimer formation (d) and 3-fold (t) and 4-fold (f) symmetry axes. Identical amino acids are indicated by asterisks. Gaps indicated by dashes were inserted to optimize the alignment. Amino acids associated with iron nucleation sites and ferroxidase activity were underlined and double-underlined, respectively. Sequence data (GenBank accession data) were as follows: feline H (AB193257) and L (AB193258), canine H (AB175610) and L (AB175612), horse H (AY112742) and L (AB175617), bovine H (AB003093) and L (AB003094), human H (NM_002032) and L (NM_000146), rabbit L (X07830), rat H (U58829) and L (NM_022500), mouse H (NM_010239) and L (J04716). Amino acid sequences of cetacean ferritin H and L subunits are from *P. crassidens*, *L. obliquidens*, *G. griseus*, and *G. macrorhynchus*.

Two or more cDNA (~4 clones) clones were sequenced for each ferritin subunit with the exception of the L subunits of *L. obliquidens *and *T. truncatus*, for which only one clone each was sequenced. Mammalian ferritin may undergo modification by release of the initiating Met and acetylation of the next residue (Ser or Thr) [18-22]. We suggest that dolphin ferritin may be subjected to the same modifications. Figures 1 and 2 show the nucleotide sequences and predicted amino acid sequences of *P. crassidens*. Nucleotide sequences of the H subunits of the six different dolphin species contained a 28-bp 5'-untranslated region followed by a 182-amino acid protein (excluding the N-terminal methionine) and a 55-bp 3'-untranslated region. Nucleotide sequences of the L subunits contained a 15-bp 5'-untranslated region followed by a 174-amino acid protein (excluding the N-terminal methionine), and a 113-bp 3'-untranslated region. Sequences of the L subunit coding regions, including the 5'- and 3'-untranslated regions were determined in *P. crassidens*, *G. griseus*, *G. macrorhynchus*, and *T. truncatus *of the six different dolphin species. However, the 3'-untranslated region of *L. obliquidens*, and both the 5'- and 3'-untranslated regions of *D. leucas *could not be determined because sufficient amounts of PCR product for sequencing could not be produced for unknown reason. Nucleotide sequences of the H subunit were highly conserved among the six different dolphin species. Substitutions as compared with *P. crassidens *in Figure 1 were as follows: G480A in *G. macrorhynchus*; T255C and T492C in *G. griseus*; a(-6)c, T408G, G504A, and G528T in *L. obliquidens*; a(-6)c, C18T, C165T, G363A, T408G, and G528T in *T. truncatus*; A177G, C182G, C208T, A228G, T255C, C400A, G449T, T450G, G459A, G480T, G528C, C534T, and c588t in *D. leucas*. Amino acid sequences of the H subunit were identical in five of the six dolphin species, the exception being *D. leucas *with three amino acid substitutions (S56C, L129I, and G145V), resulting in 98% sequence identity as compared to the canonical cetacean H subunit sequence shown in Figure 3.

Nucleotide sequences of the L subunits among the six different dolphin species were also highly conserved when compared to the *P. crassidens *sequence shown in Figure 2. The 5'-untranslated regions of the L subunits sequenced were identical, and the 3'-untranslated regions that were sequenced were identical except for one substitution (t599 g). The L subunit coding region is also markedly conserved. Substitutions as compared with *P. crassidens *were as follows: no substitution in *G. macrorhyncus*; T522C in *L. obliquidens*; G21A and T522C in *G. grisseus*; C73T, C100T, G482T, and T522C in *T. truncatus*; G184A, G325C, C510T, and T522C in *D. leucas*. Amino acid sequences of the L subunits of *P. crassidens*, *L. obliquidens*, *G. griseus*, and *G. macrorhynchus *were identical. *T. truncates *is 99% identical with one substitution (G160V), and *D. leucas *is 98% identical with two substitutions (G61S and E108Q), as compared with the canonical cetacean L subunit (*P. crassidens*, *L. obliquidens*, *G. griseus*, and *G. macrorhynchus*) shown in Figure 3. Amino acid identities between the H and L subunits of *P. crassidens*, *T. truncatus*, and *D. leucas *were 51, 50, and 51%, respectively.

Amino acid sequences of the H and L subunits of *P. crassidens*, *L. obliquidens*, *G. griseus*, and *G. macrorhynchus *were designated as canonical cetacean ferritin sequences and aligned with other known mammalian ferritin H and L subunits (Figure 3). Seven amino acids (E23, Y30, E57, E58, H61, E103, and Q137)[2,17] associated with the ferroxidase center were perfectly conserved in all mammalian H subunits including cetaceans. Residues in the iron nucleation site (H49, R52, E53, E56, E57, R59, E60, R64, K67, E136, K139, K142, K172, H173, and D174)[2,8,17] were highly conserved in mammalian L subunits, with cetaceans containing only one substitution (R59H). Residues involved in dimer formation and the 3-fold axis (iron channel) are highly conserved in amino acid character even in the presence of substitutions for cetacean H and L subunits. In the 4-fold axis, the H and L subunits may be functionally different as the hydrophobic Leu 169, present in all L subunits except for mouse, is substituted for the hydrophilic amino acid His in all H subunits. A salt bridge formed between K58 and E103 in all L subunits provides physical and chemical stability of the ferritin molecule despite the loss of ferroxidase activity [2,8,23]. Cetacean L subunits do not have the extra octapeptide found in rat and mouse L subunits. Amino acid sequence identities among mammalian H subunits are high (91–97%), and those among L subunits also high (78–91%), with cetacean H and L subunits showing especially high identity with canine and feline H and L subunits (97 and 96% in canine and feline H subunits, respectively, and 91% in canine and feline L subunits) (Table 1).

**Table 1:** Amino acid sequence identities between mammalian ferritin subunits

Subunit type	1	2	3	4	5	6	7	8	9	10	11	12	13	14	15	16	17
1: Cetacean ferritin H^1^		96	97	91	91	93	93	94									
2: Feline ferritin H			98	91	92	95	95	95									
3: Canine ferritin H				92	92	96	96	97									
4: Equine ferritin H					88	91	92	92									
5: Bovine ferritin H						91	92	92									
6: Human ferritin H							94	95									
7: Rat ferritin H								99									
8: Mouse ferritin H																	
9: Cetacean ferritin L^1^	51									91	91	88	90	84	86	78	79
10: Feline ferritin L		52									96	90	93	86	91	86	84
11: Canine ferritin L			52									92	90	86	89	85	82
12: Equine ferritin L				51									86	84	87	84	79
13: Bovine ferritin L					53									83	86	83	83
14: Human ferritin L						56									85	80	78
15: Rabbit ferritin L																85	84
16: Rat ferritin L								50									92
17: Mouse ferritin L									50								

^1^Amino acid sequences of cetacean ferritin H and L subunits were from *P. crassidens*, *L. obliquidens*, *G. griseus*, and *G. macrorhynchus*.

### The IRE sequence of dolphin H subunits

**Figure 4:**
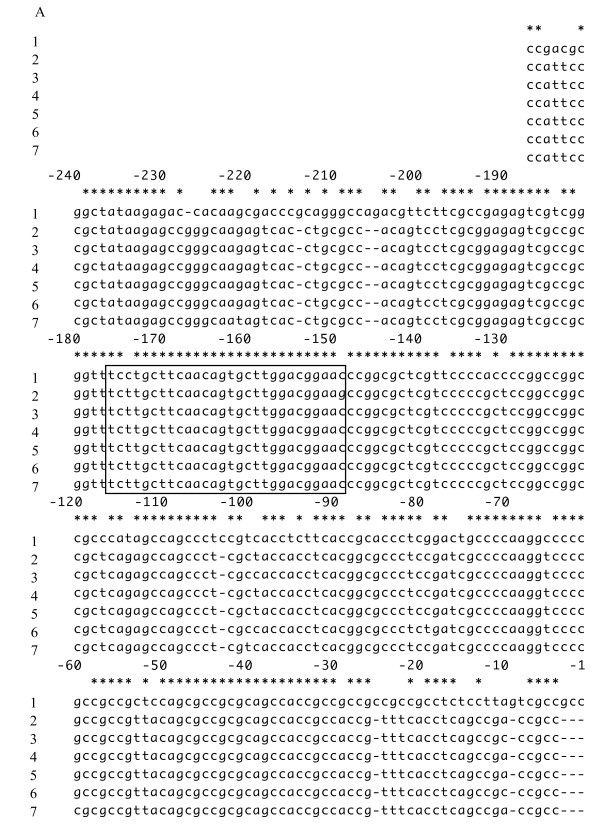
**Alignment of nucleotide sequences of the 5'-untranslated dolphin H gene with that of the human 5'-untranslated region. **IRE sequence (28 bp) was boxed. Nucleotide sequence numbering is derived from the human sequence (AF139813). Gaps indicated by dashes were inserted to optimize the alignment. 1: Human, 2: *P. crassidens*, 3: *L. obliquidens*, 4: *G. griseus*, 5: *G. macrorhynchus*, 6: *T. truncatus*, 7: *D. leucas*.

The H subunit 5'-untranslated region nucleotide sequences of the six different dolphin species were aligned with the corresponding region of the human H subunit gene (AF139813)[24](Figure 4). The boxed cetacean IRE sequences have one substitution, except for *P. crassidens *with two substitutions, as compared to the human IRE sequence. The predicted secondary structure of the cetacean IRE and its flanking sequences is similar to that of the human IRE based on the previous report (data not shown). The hairpin loop of these conserved IRE regions contains a terminal hexaloop sequence with CAGUGU and conserved UGC/C which distorts the stem loop; these regions are known to contribute to IRE-BP binding [24].

## Discussion

Although a partial ferritin subunit sequence of the seal *Phoca vitulina *(AF246195, unpublished data) has been determined, the entire coding sequences of ferritin subunits in marine mammals were previously unknown. The present study shows the ferritin coding sequences coding for the H and L subunits of six different dolphin species (*P. crassidens*, *L. obliquidens*, *G. griseus*, *G. macrorhyncus*, *T. truncatus*, and *D. leucas*). The predicted secondary structures using the GENETYX-MAC computer program (Robson method) of dolphin ferritin H and L subunits show A, B, C, D, and short E-helices and the L loop connecting B and C helices (data not shown) as in other mammalian species [1-3,18]. Sequence comparisons between dolphin ferritin and that of other mammalian species strongly suggest similar functions for the H subunit unique ferroxidase [2,3,18], the L subunit salt bridge [2,8,23] and iron nucleation site [2,8], subunit dimer formation [8,18], and the iron channel [8,18]. A putative glycosylation site was found and is conserved in both H and L subunits of mammalian ferritin, including dolphin ferritin. However, at present, glycosylated ferritin has only been found in humans [25]. Amino acid sequences of the H and L subunits among the six different dolphin species were highly conserved, but *D. leucas *is more divergent as compared with the other species, most likely because *D. leucas *is a member of the Monodontidae, whereas the others are members of the Delphinidae.

Interestingly, sequence comparisons among mammalian ferritins showed that cetacean (dolphin) is closest to carnivore (canine and feline). Cetacea (whales, dolphins, and porpoises) are believed to have evolved from Mesonychia according to dental characters [26,27]. However, phylogenic analyses based on molecular and morphologogical data have shown that Hippopotamidae are the closest extant relatives of Cetacea, and that Hippotamidae and Cetacea are closely related to the Artidactyla (cows, camels, and pigs) [26-31]. On the other hand, comparative genomic analyses that take into account genome organization (rearrangement, exchange, gene order, and association) have inferred varied rates of genome evolution among mammalian orders [32]. These rates have been found to be slow in certain species of mammals, from several different orders, including feline, mink, ferret, dolphin, and human [32]. Putative ancestral chromosomes consist of homologs of human chromosomes 11+15+19q [32]. Interestingly, human ferritin H and L subunit genes are on chromosomes 11 and 19q3, respectively [33]. This finding suggests that ferritin subunit sequences are highly conserved in mammals, and may therefore provide insufficient phylogenetic information to resolve the relationships of mammalian orders.

Cetaceans get their nutrition mainly from fish and other animal protein, as do carnivores. The iron concentration in the liver of striped dolphin (*Stenella coerulealba*) is relatively high (200 ± 12 μg/g wet weight) [34], and the iron content of ferritin prepared from the spleen of common dolphin (*Delphinus cetacea*) is also relatively high (Fe/protein ratio = 0.21) when compared to that of tuna spleen (0.14)[35]. The number of amino acids (182) in cetacean H subunits is the same as in canine and feline, and the C-terminal region shows high identity to that of canine and feline. This is significant since the ferritin iron uptake rate depends on the C-terminal region of the H subunit [36,37]. The L subunit is responsible for taking up more iron by adjusting the microenvironment within the ferritin complex [3,8,11]. Although whether or not the sequence evolution of ferritin may have been affected by factors such as body iron stores, food, or other environmental factors remains to be clarified, further study is needed to determine the sequences of ferritin subunits from Hippotamidae (hippopotamuses) which are phylogentically close to Cetaceca. In addition, the 15 amino acids involved in iron nucleation in dolphin ferritin are highly conserved, with only one substitution (R59H). However, histidine is similar to arginine as basic amino acid. Further study need to examine whether this substitution may be unique to marine mammals or not.

Ferritin synthesis is regulated at both the transcriptional and translational levels by oxidative stress and cellular iron conditions, respectively [3,4,15-17]. In order to determine if an IRE sequence was present, dolphin leukocyte genomic DNA was used because the stem loop formed by the IRE hinders the advance of the RT reaction for first-strand cDNA synthesis from mRNAs [15]. The presence of the IRE in dolphin mRNA was reveled by sequence analysis of the 5'-untranslated region of the H subunit gene, and its predicted secondary structure is similar to that of the human IRE. The dolphin ferritin H subunit IREs had conserved a base pair formed between the G in the UGC bulge and the C in the other strand unique to other mammalian ferritin IREs in addition to canonical CAGUGN loop sequences [38]. Therefore, we surmise that cetacean H and L subunits are synthesized iron-dependently at the translational level as in other mammalian species [3,15,24]. Additionally, the ARE is responsible for up-regulating ferritin expression in response to oxidative stress in order to segregate cellular Fe^2+ ^to prevent iron-catalyzed reactive oxygen species through the Fenton reaction [4,16,17]. Further study is needed to locate putative AREs expected to be found far upstream of the dolphin ferritin subunit genes.

The sequence data reported in this article has been submitted to the DNA Data Bank of Japan (DDBJ) and appears in the DDBJ, EMBL, and GenBank data banks with the following accession numbers (order of the numbers are *P. crassidens*, *L. obliquidens*, *G. griseus*, *G. macrorhynchus*, *T. truncatus*, and *D. leucas*). H subunit: AB371409, AB371412, AB371415, AB371418, AB371421, AB371424; L subunit: AB371411, AB371414, AB371417, AB371420, AB371423, AB371426; 5'-untranslated region of H subunit: AB371410, AB371413, AB371416, AB371419, AB371422, AB371425.

## Conclusion

Sequence analyses of cetacean ferritin H and L subunits were performed by direct sequencing of PCR fragments from cDNAs generated via RT-PCR of leukocyte total RNA prepared from blood samples of six different dolphin species (*Pseudorca crassidens*, *Lagenorhynchus obliquidens*, *Grampus griseus*, *Globicephala macrorhynchus*, *Tursiops truncatus*, and *Delphinapterus leucas*). Dolphin H and L subunits consist of 182 and 174 amino acids, respectively, with each subunit showing high sequence identity with the corresponding subunit of mammalian ferritin (H: 91–97%; L: 78–91%). The putative iron-responsive element sequence in the 5'-untranslated region of the six different dolphin species was demonstrated by direct sequencing of PCR fragments obtained using each leukocyte genomic DNA.

## Competing interests

The authors declare that they have no competing interests.

## Authors' contributions

AT, KW, and KO participated in the discussion on the study design, the collection of dolphin blood samples, analysis of sequence data, and the writing manuscript. AT, ST and YK performed sequencing of cDNA clones and PCR fragments. All authors read and approved the final manuscript.
